# Supplementary material for: Identifying putative ventilation-perfusion distributions in COVID-19 pneumonia
Source: PLoS One. 2022 Aug 30;17(8):e0273214. doi: 10.1371/journal.pone.0273214 (PMC9426945; doi:10.1371/journal.pone.0273214)
Supplement: Attachment — Submitted filename: response_to_reviewers_08032022.docx [file pone.0273214.s002.docx]

# Responses to Reviewers’ Comments

Dear Editor

Thank you very much for the return of our manuscript with additional points for revision. Our responses to the reviewers are given in italic to each of their comments (below). We hope this is now suitable for publication.

Yours sincerely

Peter Robbins

**Submitted title**

Identifying putative ventilation-perfusion distributions in COVID-19 pneumonia

**Respond to reviewers’ comments**

**Reviewer #1**

The paper improved after a significant revision that partially incorporated the major and minor questions. I think the paper has merit, even if some major points persist and must be thoroughly addressed.

**Comment 1**

I am very confused by the use of the words “solution”, “model”, and “problem”. Mathematically speaking, a solution is a value, number, function, etc… that fulfill the conditions set by equations. Here, I find very hard to understand which are the equations to be solved (models and problems) and which variables are the solutions by reading through the text. Figure 1 is adding confusion rather than giving a clear picture of the problems/equations and solutions searched for. What is meaning that a “solution is not possible”? I also find very difficult to identify data and solutions (?) in the models.

**Response to comment 1**

*Thank you – this is very helpful comment to guide further revision. We have added a paragraph at the beginning of the Methods to define the terms ‘solution’, ‘model’ and ‘problem’.*

*A solution is a set of paired values for ventilation and perfusion {Vi, Qi}, that will result in calculated values for arterial PCO2 and PO2 that, to within experimental error, match the measured values from the patient. We illustrate solutions in the figures. The meaning of a ‘solution is not possible’ is that, after applying constraints relating to the patient, there are no sets {Vi, ,Qi} that form a solution to the problem. This can arise if one or more of the constraints is wrong.*

**Comment 2**

The concept of “distribution” should be introduced and carefully presented. If I understand correctly, this work revolves around the idea of calculating such distribution instead of a sort of “trial and error” approach by Busana et al., who explore the space of plausible parameters to identify a plausible combination of these ones. Can this distribution interpreted instead as a combination of values?

**Response to comment 2**

*We have added a definition of distribution in the paragraph at the start of the Methods. Busana et al do not state what their parameters are, but basically their parameters are used to generate the ‘ V/Q distribution’, which is the set of paired values {Vi, Qi}. The number of paired values in this set can be varied. We explore the three-compartment model of the lung – which has three pairs of values in this set (and has a very important place in the development of theory around gas exchange in the lung), a four-compartment model of the lung, and a ‘multi’-compartment model of the lungs, where the number of Vi, Qi pairs is very much higher and where it is really being used as a computational approximation to a continuous distribution (where the pairs of values Vi, Qi in the set {Vi, Qi} would be infinite).*

*So the answer to your question is yes. The distribution is the set {Vi, Qi}.*

**Comment 3**

118: “conservation of matter”. Shouldn’t be conservation of mass instead? Here, we are not working in the framework of relativistic mechanics, but of classical mechanics.

**Response to comment 3**

*We have changed this.*

**Comment 4:** 150: Eq. (5). g is not defined, if not much later in the text. Similarly for other mathematical notation.

**Response to comment 4**

*As indicated in the preceding paragraph, g is a function that represents the blood gas model of reference 7. To make this clearer, we have now added the reference in the sentence immediately preceding equation 5.*

**Comment 5**

Equations are numbered in round brackets (XY). However, in the text they are referred to as Eq XY. Instead, bibliographic references are cited in round brackets (in place of more common squared brackets), which is adding confusion to the “Method” section.

**Response to comment 5**

*We have revised the manuscript so that the citations now appear in the text in squared brackets.*

**Comment 6**

356-365: are these part of the table caption?

**Response to comment 6**

*Yes, that is correct.*

**Reviewer #2**

It is hard to evaluate the changes made by the authors. They should clearly indicate in their answers the number of pages and lines where changes have been made and, possibly, indicate in colours such changes in the text.

**Response to reviewer #2**

*Our apologies for this. We used the track changes feature to highlight all the text that had changed, but there was so much of it, and so much editing, that the end result was a bit of a mess. The changes are much clearer in this second revision.*
